# Upweighting rare favourable alleles increases long-term genetic gain in genomic selection programs

**DOI:** 10.1186/s12711-015-0101-0

**Published:** 2015-03-21

**Authors:** Huiming Liu, Theo HE Meuwissen, Anders C Sørensen, Peer Berg

**Affiliations:** Center for Quantitative Genetics and Genomics, Department of Molecular Biology and Genetics, Aarhus University, P. O. Box 50, 8830 Tjele, Denmark; Department of Animal and Aquacultural Sciences, Norwegian University of Life Sciences, P. O. Box 5003, 1432 Ås, Norway; Nordic Genetic Resource Center, P. O. Box 115, 1431 Ås, Norway

## Abstract

**Background:**

The short-term impact of using different genomic prediction (GP) models in genomic selection has been intensively studied, but their long-term impact is poorly understood. Furthermore, long-term genetic gain of genomic selection is expected to improve by using Jannink’s weighting (JW) method, in which rare favourable marker alleles are upweighted in the selection criterion. In this paper, we extend the JW method by including an additional parameter to decrease the emphasis on rare favourable alleles over the time horizon, with the purpose of further improving the long-term genetic gain. We call this new method dynamic weighting (DW). The paper explores the long-term impact of different GP models with or without weighting methods.

**Methods:**

Different selection criteria were tested by simulating a population of 500 animals with truncation selection of five males and 50 females. Selection criteria included unweighted and weighted genomic estimated breeding values using the JW or DW methods, for which ridge regression (RR) and Bayesian lasso (BL) were used to estimate marker effects. The impacts of these selection criteria were compared under three genetic architectures, i.e. varying numbers of QTL for the trait and for two time horizons of 15 (TH15) or 40 (TH40) generations.

**Results:**

For unweighted GP, BL resulted in up to 21.4% higher long-term genetic gain and 23.5% lower rate of inbreeding under TH40 than RR. For weighted GP, DW resulted in 1.3 to 5.5% higher long-term gain compared to unweighted GP. JW, however, showed a 6.8% lower long-term genetic gain relative to unweighted GP when BL was used to estimate the marker effects. Under TH40, both DW and JW obtained significantly higher genetic gain than unweighted GP. With DW, the long-term genetic gain was increased by up to 30.8% relative to unweighted GP, and also increased by 8% relative to JW, although at the expense of a lower short-term gain.

**Conclusions:**

Irrespective of the number of QTL simulated, BL is superior to RR in maintaining genetic variance and therefore results in higher long-term genetic gain. Moreover, DW is a promising method with which high long-term genetic gain can be expected within a fixed time frame.

**Electronic supplementary material:**

The online version of this article (doi:10.1186/s12711-015-0101-0) contains supplementary material, which is available to authorized users.

## Background

A number of alternative approaches have been proposed for genome-based prediction (GP) of genetic values, i.e. genomic estimated breeding values (GEBV), and many studies have focused on improving the prediction accuracy and short-term genetic gain using different approaches [1-4]. These approaches differ mainly with respect to the assumptions made on marker effects and the shrinkage method used. For instance, genomic best linear unbiased prediction (GBLUP) gives equal *a priori* weight to all markers. In GBLUP, the degree of shrinkage is marker-effect independent but frequency dependent: there is less shrinkage towards 0 for markers with intermediate frequencies [5,6]. Under this mechanism, the genetic selection differential can be maximized largely due to those markers [6]. In some Bayesian methods, e.g. Bayesian Lasso (BL), the assumption of common prior variance is relaxed, and markers with large effects are shrunk less strongly towards 0 [5]. Under this assumption, the increase in the genetic selection differential is largely due to changes in allele frequencies at markers with large effects.

Maximizing the current genetic selection differential results in the maximization of short-term genetic gain. However, repeated use of this selection procedure does not necessarily maximise long-term genetic gain over a longer time horizon, which is the goal of most breeding schemes [7]. Simulation studies that assume that quantitative trait loci (QTL) are known have applied optimal control theory [8] to maximize the long-term genetic gain [9-11]. However, maximization of long-term genetic gain in genomic selection based on marker panels is not well understood [7]. Strategies for the maximization of long-term genetic gain are different from those for the maximization of short-term genetic gain. Although a QTL with a small effect and/or with a low frequency of the favourable allele may not be important for short-term gain, it potentially contributes more to long-term genetic gain by maintaining genetic variance over time. Hence, over a longer time horizon, these alleles should be maintained in the population, for example by upweighting them in the selection criterion. Goddard [12] proposed an optimal index that is expected to maximize the long-term genetic gain with a two-QTL model example. It was suggested that, in the genomic selection model, the optimum weight for each marker depends on its allele frequencies, such that a marker with a high (low)-frequency of the favourable allele obtains a low (high) weight in the index. Marker effects were not included in this index. Goddard’s optimization was further implemented by Jannink [13], however, marker effects as well as allele frequencies were included in the selection criterion, since it is uncertain how accurately the marker effects are estimated and whether the alleles are favourable or not. Also, when there are many genes (compared to Goddard’s two-loci example), it makes sense to prioritize the loci according to their predicted effect in order to counteract random drift where it matters most. Jannink [13] showed that, as expected from Goddard [12], selection on this index initially resulted in lower accuracy of selection and genetic gain than selection on unweighted GP. However, markers close to QTL remained polymorphic much longer when selection was on the index, leading to greater genetic variance and a further improvement in genetic gain in later generations [13].

The studies of Goddard [12] and Jannink [13] assumed that selection was performed for a sufficient amount of time to fix all favourable alleles. However, when making decisions for optimum selection, the end of the time horizon might be prior to a selection limit [14]. If the time horizon is short, increased emphasis on rare favourable alleles is no longer essential to increase genetic gain, and therefore, short-term genetic gain should be maximized. To this end, we hypothesize that long-term genetic gain can be maximized by gradually decreasing weights on the rare favourable alleles as the population approaches the end of the time horizon. Also, Goddard’s optimization [12] and Jannink’s implementation [13] assume that marker effects are known without error and that markers are in complete linkage disequilibrium (LD) with QTL. However, Bijma [6] argued that even if the true effects of alleles are known and selection is for the optimal combination of all true allele effects, drift should be accounted for because of Mendelian sampling, linkage and recombination. Thus, by chance certain favourable alleles will inevitably be absent in the selected individuals. For this reason, Bijma [6] argued that the optimum weights on rare favourable alleles should be greater than the optimum weights of Goddard [12]. By doing so, rare favourable alleles would be rapidly selected towards higher frequency, thus reducing the probability of losing them from the population.

In this study, we simulated a long-term breeding program with a time horizon of 15 or 40 generations, in order to test the following four hypotheses: (1) different GP approaches impact long-term genetic gain because of the different shrinkage methods used; to test this, ridge regression (RR) under a Bayesian framework was compared to BL for GP; (2) long-term genetic gain from RR and BL can be enhanced by weighting marker effects as a function of favourable allele frequencies (Jannink’s weighting); (3) an alternative weighting method that accounts for the time horizon of selection (dynamic weighting) can further improve the long-term genetic gain, with small reductions of short-term genetic gain; and (4) a dynamic weighting method is able to increase long-term genetic gain within different time horizons. In parallel, genetic variance, accuracy of selection, inbreeding and loss of favourable alleles over generations were also studied, which helped to interpret the consequences of the different strategies.

## Methods

### Scenarios

Different time horizons, 15 generations (TH15) or 40 generations (TH40), were used to test the sensitivity of the assumption on time horizon. Varying numbers of QTL were used, ranging from 25 to 500 per chromosome. Thus, six scenarios were analyzed (Table 1). Selection criteria included unweighted GEBV, weighted GEBV using Jannink’s weighting (JW) [13], and weighted GEBV using dynamic weighting (DW).

**Table 1:** **Summary of scenarios with respect to number of QTL per chromosome with a time horizon of 15 generations**

**Scenarios**	**Number of QTL per chromosome**	**Total QTL effects (se)**
100QTL_TH15	25	5.04 (0.043)
400QTL_TH15	100	10.04 (0.042)
2000QTL_TH15	500	22.20 (0.033)

The scenarios under time horizon of 40 (100QTL_TH40, 400QTL_TH40 and 2000QTL_TH40) have the same mean and standard errors of total QTL effects as the scenarios with the same number of QTL under time horizon of 15.

### Selection criteria

#### Unweighted genomic prediction

Two genetic models, RR and BL, were used for unweighted genomic prediction. The marker effects were estimated using the following linear model:

$$ {y}_i=\mu +{\displaystyle {\sum}_{j=1}^p}{x}_{ij}{\beta}_j+{e}_i, $$

for animal *i* (*j* = 1, 2 …, *p* markers), where *y*_*i*_ is the phenotypic records, *μ* is the intercept, *x*_*ij*_ is the marker covariate (0, 1 or 2), and ${\left\{{\boldsymbol{\beta}}_{\boldsymbol{j}}\right\}}_{\boldsymbol{j}=\mathbf{1}}^{\boldsymbol{p}}$ is a vector of marker effects. The breeding value *g*_*i*_ for unweighted GP was defined as ${g}_i={\displaystyle {\sum}_{j=1}^p}{x}_{ij}{\beta}_j.$ Gaussian assumptions for model residuals were applied, i.e. the joint distribution of model residuals was assumed to follow $\mathrm{N}\left(0,{\upsigma}_{\mathrm{e}}^2\right)$. The likelihood function yields:

$$ p\left(y\Big|\mu, g,\ {\upsigma}_{\mathrm{e}}^2\right) = {\displaystyle \prod_{\mathrm{i}=1}^{\mathrm{n}}}\mathrm{N}\left({y}_i\Big|\mu +{\displaystyle \sum_{j=1}^p}{x}_{ij}{\beta}_j,{\upsigma}_{\mathrm{e}}^2\right), $$

where $\mathrm{N}\left({\mathrm{y}}_{\mathrm{i}}\Big|\mu +{\displaystyle {\sum}_{j=1}^p}{x}_{ij}{\beta}_j,{\upsigma}_{\mathrm{e}}^2\right)$ is a normal density for random variable *y*_*i*_ centered at $\mu +{\displaystyle {\sum}_{j=1}^p}{x}_{ij}{\beta}_j$ and with variance ${\upsigma}_{\mathrm{e}}^2$ [15]. Ridge regression and BL differ in the prior distribution for marker effects. For RR, a common variance is assigned to all marker effects, i.e. ${\beta}_j\sim N\left({\beta}_j\Big|0,\ {\sigma}_{\beta}^2\right),$ where ${\sigma}_{\beta}^2$ is the prior variance of marker effects. For BL, marker-specific variance is assigned to individual marker effects, i.e. ${\beta}_j\sim DE\left({\beta}_j\Big|0,\frac{\lambda }{\sigma_e^2}\right),$ where DE is the double exponential (Laplace) distribution [15], *λ* is a regularization parameter, and ${\sigma}_e^2$ is the prior variance of random residuals.

Gianola [5] has shown that RR does not lead to uniform shrinkage of marker effects. The weight assigned to marker *j* is:

$$ {W}_j=\frac{{\displaystyle {\sum}_{i=1}^n}{x}_{ij}^2}{{\displaystyle {\sum}_{i=1}^n}{x}_{ij}^2+\lambda}\approx \frac{2{p}_j\left(1-{p}_j\right)}{2{p}_j\left(1-{p}_j\right)+\frac{\lambda }{n}}, $$

where *p*_*j*_ is the frequency of one of the alleles at locus *j*, $\lambda =\frac{\upsigma_{\mathrm{e}}^2}{\upsigma_{\upbeta}^2}$ , and n is the number of animals [5]. This shows that the extent of shrinkage in RR is frequency and sample-size dependent. It is clear that, for a fixed sample size n, there is less shrinkage towards 0 for markers with intermediate allele frequencies, irrespective of the effect. In contrast, BL puts a higher weight on markers with a larger effect, which is reflected by the shrinkage factor $\frac{\sigma_e^2\lambda }{\left|{\beta}_j\right|}$, where λ is a regularization parameter that controls the prior on *β*_*j*_ [5,16].

For RR, a scaled inverse *χ*^2^ distribution with degrees of freedom (df) equal to 4 and scale equal to 1 was assigned to ${\sigma}_e^2$ and ${\sigma}_{\beta}^2$. For BL, the residual variance was the same as for RR, and the rate and shape parameters for $\boldsymbol{\uplambda}$ were set to be 1 × 10^−4^ and 0.6 respectively, following the guidelines in [17]. Marker effects were estimated using RR and BL, as described in [15,18] and as implemented in the R-package BLR [19]. The Gibbs sampler was run for 1500 iterations and the first 500 iterations were discarded as burn-in for both RR and BL. Details on the model and algorithms are in [10,15].

### Weighted genomic prediction

For weighted GP, the marker effects *β*_*j*_ were initially estimated by RR or BL as described above. Frequencies of the favourable allele were also calculated for all markers. The favourable (unfavourable) allele of each marker was determined by the positive (negative) sign of the estimated marker effect. More specifically, the marker covariates were coded as 0, 1 or 2, as for the estimation of marker effects. The code counts the number of copies of one of the alleles (“1” or “2”) observed at a locus. If the number of allele “1” was counted and the sign of the estimated marker effect was positive, then “1” was taken as the favourable allele. Then, the weight used for each marker in JW was:

$$ {w}_{Jj}=\frac{\left[ \arcsin (1)- \arcsin \left(\sqrt{p_j}\right)\right]}{\sqrt{p_j\left(1-{p}_j\right)}}, $$

where *w*_*Jj*_ is the weight for marker *j* based on [13], and *p*_*j*_ is the favourable allele frequency for marker *j*. Thus, the weight given to each SNP depended on the favourable allele frequency which changes in each generation. The assumption behind this weighting scheme is that selection is performed for a sufficient amount of time to fix all favourable alleles. The expression reflects the total accumulated selection intensity required to move the allele to fixation. The objective is to fix high-frequency favourable alleles of markers almost at the same time as low-frequency favourable alleles.

**Figure 1:**
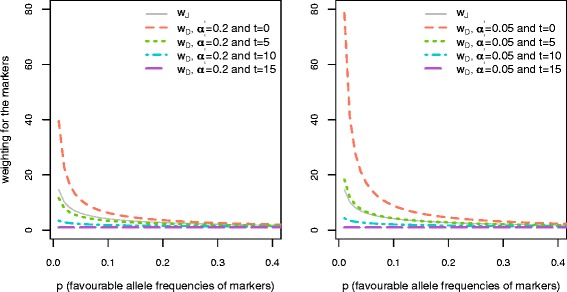
**Weighting on markers given favourable marker allele frequencies in Jannink and dynamic weighting (** ***w***_***J***_ **and** ***w***_***D***_ **).** The time horizon is 15 generations, and t is the generation number. Dynamic weighting in the left plot is under *α* = 0.2 and that in the right plot is under *α* = 0.05. Note that weights were scaled by dividing the minimal possible value (given a favourable allele frequency (p) equal to 0.999), so the minimum value for all weights in generation t becomes 1. When the frequency of markers approaches 0.4, the weight is almost equivalent to 1, thus those markers are not included in the figure.

For DW, in order to allow the weighting to vary according to time horizon, we used the probability density function of the beta distribution:

$$ {W}_{Dj}= Beta\left(\alpha,\ b\right): prob\left(p\Big|\alpha,\ b\right)=\frac{{p_j}^{\alpha -1}{\left(1-{p}_j\right)}^{b-1}}{B\left(\alpha, b\right)}, $$

where *w*_*Dj*_ is the weight for marker *j* based on dynamic weighting, *α* and *b* are shape parameters, and *B* is the beta function. With this function, two aspects are incorporated in DW. First, the initial weighting on markers with a low favourable allele frequency is higher than in JW. In other words, more weight is allocated to favourable alleles with a low frequency compared to JW, to ensure that the rare alleles are maintained. For instance, at G_0_, if *α* is set to 0.2, *b* to 1, and the favourable allele frequency *p* to 0.05, the weighting given to this marker would be 17.21 times higher than the maker with a *p* of 0.999. In contrast, with JW, the weighting on that marker would be only 6.17 times higher than the maker with a *p* of 0.999. Second, the weighting can be adjusted according to the remaining number of generations of selection. For DW, we introduced “current generation” and “total time horizon” into the above probability density function:

$$ {W}_{Dj}= Beta\left(\alpha,\ b\right): prob\left({p}_j\left|\right(\alpha ={\alpha}^{\hbox{'}}+t*\frac{\left(1-{\alpha}^{\hbox{'}}\right)}{N},\ b=1\right)=\frac{{p_j}^{\left({\alpha}^{\hbox{'}}+t*\frac{\left(1-{\alpha}^{\hbox{'}}\right)}{N}\right)}}{B\left(\alpha, b\right)}, $$

where *α*' is a parameter that determines the initial weight assigned to the markers, *t* is the generation when the weight is assigned to each marker, and *N* is the generation for which the total long-term genetic gain is to be maximized. According to this function, as ${\alpha}^{\hbox{'}}+t*\frac{\left(1-{\alpha}^{\hbox{'}}\right)}{N}$ approaches 1 (t tends to N), the weight assigned to each marker approaches 1, which indicates that selection is aimed at maximizing the short-term genetic gain. In other words, maintaining favourable alleles with a low frequency becomes less important as the end of the selection program is reached. Parameter *α*^'^ was set to 0.05 or 0.2 for comparison and to evaluate the importance of putting a higher weight on the rare favourable alleles compared to JW. The weighting as a function of favourable allele frequencies and generations for TH40 is in Figure 1. As a result, the estimated breeding value of each individual was calculated as:

$$ {g}_i={\displaystyle {\sum}_{j=1}^p}{x}_{ij}{\beta}_j{w}_{J(D)j}, $$

where *w*_*J*(*D*)*j*_ is the weight for marker *j* based on Jannink’s weighting or dynamic weighting. As in [13], marker effects *β*_*j*_ were included in the expression of the selection criterion to reduce the importance of small-effect loci for which the favourable allele could not be determined with any certainty [13].

### Simulations

#### Genome structure

A historical population with a size of 100 males and 100 females for 2000 discrete generations was simulated using QMSim [17]. The simulated genome consisted of four 1 Morgan long chromosomes, on which 10 000 loci were equally distributed, resulting in 40 000 loci across the genome. Among all simulated loci, every second locus was used as the position of a potential marker, whereas the remaining loci were used as positions of potential QTL. At generation 0, all loci were simulated to be bi-allelic with allele frequencies equal to 0.5 and alleles coded as “1” and “2”. Details regarding recombination and mutation rates are in [18]. Generation 2000 was used as the base population (G_0_). At G_0_, the average (±SD) linkage disequilibrium (LD) between neighbouring loci was r^2^ = 0.27 (±0.32), and the allele frequency distribution followed a U- shaped distribution, with ~30.2% of the loci fixed. Subsequently, the markers and QTL were chosen among all segregating loci according to their minor allele frequencies (MAF). Markers with a MAF greater than 0.05 were picked as the real markers, and a specific number of QTL, depending on the scenario, with a MAF greater than 0.01 were uniformly picked among the potential QTL. As a consequence, 8257 markers that were approximately evenly distributed across the genome were used in the selection procedures.

### Trait simulation

The simulated traits were standardized to have a mean of 0 and a variance equal to the heritability, i.e. 0.1 for animals in G_1_. Generations 1 to 25 or 40 were simulated without mutations. The QTL effects were assumed to follow a gamma distribution Γ(1.48, 0.09), following the shape parameter for distribution of QTL effects in pigs [20]. The effects of those QTL were standardized to achieve an initial genetic variance equal to the heritability (0.1 for all scenarios in this study), i.e.:

$$ {a}_j={a}_j^{\hbox{'}}*\sqrt{\frac{h^2}{{\displaystyle {\sum}_{i=1}^n}2{p}_i\left(1-{p}_i\right){a}_i^{\hbox{'}}}}, $$

where *h*^2^ is the heritability, subscripts *i* (*j*) denote QTL *i* (*j*), *p*_*i*_ (*p*_*j*_) is the frequency of the “1” allele of QTL *i* (*j*), and ${a}_i^{\hbox{'}}$ (${a}_j^{\hbox{'}}$) is the substitution effect of QTL *i* (*j*) before being scaled. The additive QTL variance explained all genetic variance. True breeding values (TBV), environmental terms and phenotypic records at each generation were simulated as described in [21].

From G_0_ to G_1_, the population size was increased from 200 to 500, i.e. 50 females that were selected based on the selection criterion in G_0_ produced 10 offspring. Selection was then continued for 15 or 40 generations. In each generation, the best 5 males and 50 females among 500 candidates were selected based on the selection criterion. Selected individuals were randomly mated and each pair produced 10 offspring with equal sex ratio. For all selection criteria, marker genotypes and phenotypes at G_t_ (generation t) and G_t-1_ were assumed to be known, and the prediction model for GP was updated every generation.

### Data analysis

For each of the scenarios, summary statistics were based on 100 replicated simulations. It should be noted that for unweighted GP and GP using JW, the simulation of TH15 was nested within TH40, since there were no additional parameters to control the time horizon. This means that the results for TH15 were reported from the data for TH40. In contrast, simulations for TH15 and TH40 were done separately for GP using DW. For each simulation, long-term genetic gains from selection were standardized by the maximal genotypic value possible for the genetic model (i.e. when all favourable QTL alleles are fixed) as follows:

$$ {R}_s=\frac{R_t}{R_m-{R}_0}, $$

where *R*_*s*_ is the long-term genetic gain after the standardization, *R*_0_ is the absolute average TBV in G_0_, *R*_*t*_ is the absolute long-term genetic gain in G_t_, which is calculated as the average TBV in G_t_ minus R_0_, and *R*_*m*_ is the maximal genotypic value that can be achieved under the current genetic model. Therefore, *R*_*s*_ represents the long-term genetic gain achieved as a proportion of the total genetic gain that can be achieved under the current genetic model, and is expressed on a 0 to 1 scale. Comparisons between criteria in terms of genetic gain were mainly based on *R*_*s*_. It should be noted that *R*_*m*_ differs among scenarios with different numbers of QTL (Table 1), i.e. *R*_*m*_ increases as the number of QTL increases. The empirical accuracy of GEBV at each generation was calculated as the correlation between TBV and GEBV. Inbreeding coefficients in G_t_, F_t_, were estimated with the inbreeding function in the R-package GeneticsPed [22], using the algorithm of Meuwissen and Luo [23] and all pedigree information from G_0_ to G_40_. The rate of inbreeding (Δ*F*_*t*_) given the inbreeding coefficient in G_t_ and the inbreeding coefficient in the base population (*F*_0_, equivalent to 0) was calculated as $\Delta {F}_t=1-\sqrt[t]{\frac{1-{F}_t}{1-{F}_0}}$, which was derived from the equation in [24]. Moreover, the Mendelian selection differential (MSD) was calculated according to the method described in Pedersen *et al.* [25]. Briefly, the Mendelian sampling term was calculated as the difference between an animal’s TBV and the mean TBV of its parents. Then, the difference between the mean Mendelian sampling terms of selected animals and all candidates was calculated and scaled by the genetic standard deviation, resulting in MSD. The difference between selection schemes with respect to genetic gain, Δ*F*, and MSD were analyzed using Tukey's HSD (honest significant difference) test, in conjunction with ANOVA (p < 0.05).

## Results

### Consequences of unweighted genomic prediction

**Figure 2:**
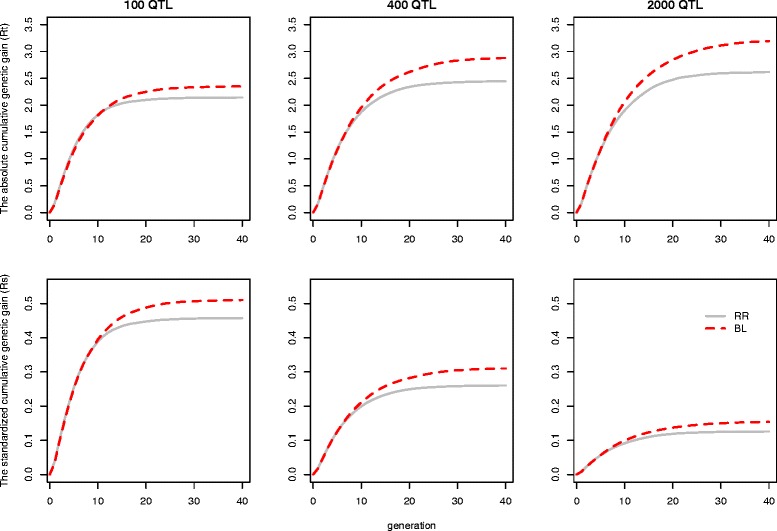
**Absolute long-term genetic gain (** ***R***_***t***_ **) and standardized long-term genetic gain (** ***R***_***s***_ **) from G**_**0**_ **to G**_**40**_ **with truncation selection on GEBV estimated with unweighted GP.**

Table 2 presents the standardized long-term genetic gain *R*_*s*_ at G_15_ or G_40_ when no weighting is applied. In general, there was no significant difference between RR and BL in *R*_*s*_ at G_1_, except for the scenario with 100 QTL. But RR resulted in significantly lower *R*_*s*_ at G_15_ and G_40_ than BL. For instance, RR provided the following *R*_*s*_ at G_15_: 43.4, 23.4 and 11.0% of the maximal genetic value with 100, 400 and 2000 QTL. With BL, *R*_*s*_ increased significantly up to 46.1, 25.9 and 12.4% respectively. Similarly, at G_40_, BL led to 11.6, 19.2 and 22.7% higher *R*_*s*_ than RR. Figure 2 shows that the superiority of BL over RR in *R*_*s*_ occurred after 8 to 10 generations of selection in all scenarios. In addition to genetic gain, BL also had a ~16.7% lower inbreeding rate at G_0_ to G_15_ than RR, irrespective of the number of QTL affecting the trait (Table 2).

**Table 2:** **Standardized long-term genetic gain after one, 15 and 40 generations of unweighted genomic selection, as percentage of the total achievable genotypic value (with fixation of all favourable alleles)** ***R***_***s***_ **, and rate of inbreeding ΔF in the first 10 generations**

**Scenario**	**Selection criteria**	***R***_***s***_ **(%)**			**ΔF**
		**G**_**1**_	**G**_**15**_	**G**_**40**_	**G**_**0**_ **-G**_**10**_
nQTL = 100	RR	3.74^a^	43.43^b^	45.74^b^	0.060^a^
	BL	3.26^b^	46.09^a^	51.06^a^	0.050^b^
nQTL = 400	RR	1.83^c^	23.35^d^	26.04^d^	0.063^a^
	BL	1.77^c^	25.94^c^	31.03^c^	0.052^b^
nQTL = 2000	RR	0.81^d^	11.00^f^	12.59^f^	0.063^a^
	BL	0.82^d^	12.35^e^	15.45^e^	0.052^b^

The standard errors for all the means are < 0.85%; different superscripts in the same column show significant differences (p < 0.05).

Increasing the number of QTL tended to increase the total absolute long-term genetic gain *R*_*t*_ (Table 1), while it significantly decreased *R*_*s*_ for both TH15 and TH40. Moreover, increasing the number of QTL increased the relative difference between BL and RR in both *R*_*t*_ and *R*_*s*_. The number of QTL, however, did not influence the rate of inbreeding (Table 2).

### Consequences of weighted genomic prediction

**Figure 3:**
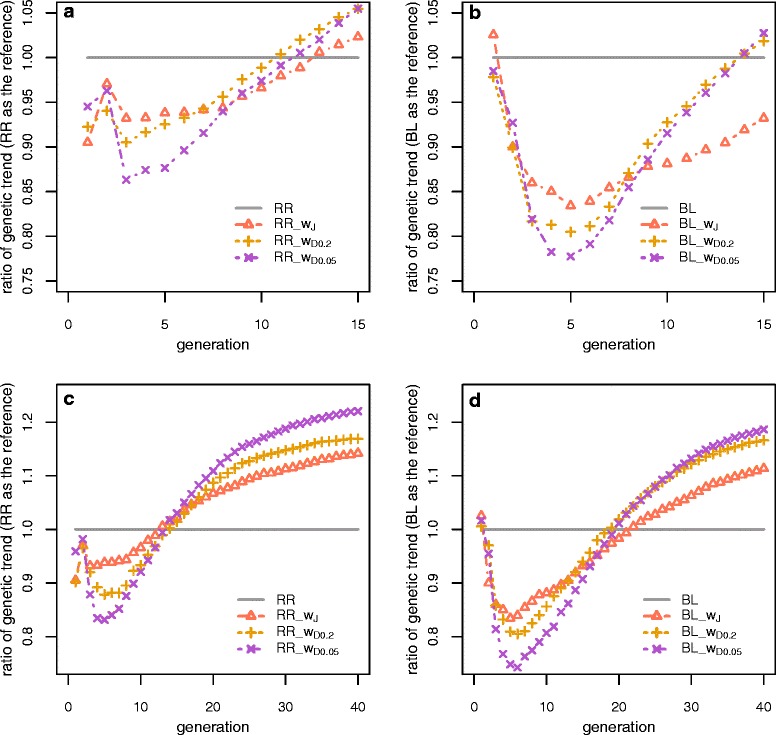
**Ratio of standardized long-term genetic gain from weighted GP to unweighted GP.** Plots a and b present the ratio of long-term genetic gain with weighted GP to that with unweighted GP under TH15, and graphs c and d present the same information under TH40.

Figure 3 shows the ratio of *R*_*s*_ using weighted GP relative to that using unweighted GP with 100 QTL in each generation. Focusing first on TH15, *R*_*s*_ was higher using unweighted GP compared to using weighted GP, except near the end of the selection program. Also, compared to with unweighted GP, with weighted GP, *R*_*s*_ initially decreased to a great extent, especially when using BL to estimate marker effects. The decrease was more pronounced with DW than with JW. After four to five generations of selection, *R*_*s*_ from weighted GP tended to catch up with that from unweighted GP. *R*_*s*_ from unweighted GP was overtaken by DW from G_11_ when using RR and from G_14_ when using BL, and consequently DW showed a 1.3 to 5.5% higher total *R*_*s*_ than unweighted GP and DW within TH15 (Table 3). Using JW, however, resulted in 6.8% lower *R*_*s*_ than using unweighted GP when BL was used to estimate the marker effects.

**Table 3:** **Standardized long-term genetic gain** ***R***_***s***_ **after one and 15 generations of weighted genomic selection with a time horizon of 15 generations**

**Scenario**	**Selection criteria**	***R***_***s***_ **(%)**			
		**G**_**1**_	**G**_**15**_	**Increased gain (%)** ^*^	**ΔF(G**_**0**_ **-G**_**10**_ **)**
n QTL = 100	RR_w_J_	3.39^a^	44.43^ab^	2.30	0.050^a^
	RR_w_D0.2_	3.46^a^	45.80^ab^	5.46	0.053^a^
	RR_w_D0.05_	3.54^a^	45.81^ab^	5.48	0.050^a^
	BL_w_J_	3.34^a^	42.96^b^	−6.79	0.037^b^
	BL_w_D0.2_	3.09^a^	46.70^a^	1.32	0.039^b^
	BL_w_D0.05_	3.15^a^	47.36^a^	2.76	0.037^b^
n QTL = 400					
	RR_w_J_	1.93^a^	25.08^a^	6.77	0.051^a^
	RR_w_D0.2_	1.67^a^	25.46^a^	8.39	0.052^a^
	RR_w_D0.05_	1.92^a^	25.91^a^	10.32	0.052^a^
	BL_w_J_	1.63^a^	23.32^b^	−9.75	0.038^b^
	BL_w_D0.2_	1.64^a^	26.27^a^	1.66	0.039^b^
	BL_w_D0.05_	1.74^a^	25.86^a^	0.27	0.038^b^
n QTL = 2000					
	RR_w_J_	0.81^a^	11.41^cd^	3.73	0.050^a^
	RR_w_D0.2_	0.74^ab^	11.83^bc^	7.55	0.054^a^
	RR_w_D0.05_	0.78^ab^	12.10^ab^	10.00	0.052^a^
	BL_w_J_	0.73^ab^	11.00^d^	−10.86	0.037^b^
	BL_w_D0.2_	0.68^ab^	12.44^a^	0.81	0.040^b^
	BL_w_D0.05_	0.68^b^	11.97^abc^	−3.00	0.037^b^

Different superscripts in the same column show significant differences (p < 0.05); standard errors for all means are for the long-term genetic gain are < 0.21% for G_1_, < 0.79% for G_15_ and for <0.01 ΔF; ^*^the increase in *R*_*s*_ from unweighted GP relative to weighting GP under the same prediction model.

When the time horizon was increased to 40 generations, a similar pattern was found, i.e. the initial decrease and later increase in *R*_*s*_ for weighted GP relative to unweighted GP. However, the increase in *R*_*s*_ was much more marked compared to that with TH15. As shown for RR in Figure 3, for instance, all weighted GP caught up with unweighted GP for *R*_*s*_ at G_13_ and G_14_ with 100 QTL. Consequently, JW increased *R*_*s*_ by 14.2%, and DW further increased *R*_*s*_ by 16.9 (RR_w_D0.2_) to 22.1% (RR_w_D0.05_), but at a cost of lower initial gain. Similarly, BL_w_J_ led to an increase in *R*_*s*_ by 11.36% relative to BL, and it was further increased to 16.63% for RR_w_D0.2_ to 18.64% for RR_w_D0.05_.

Tables 3 and 4 show that, with more than 100 QTL, the effect of weighting on *R*_*s*_ was slightly larger in relative terms. The effect of weighting on rate of inbreeding was not affected by the number of QTL.

**Table 4:** **Standardized long-term genetic gain** ***R***_***s***_ **after one and 40 generations of weighted genomic selection and rate of inbreeding with a time horizon of 40 generations**

**Scenario**	**Selection criteria**	***Rs*** **(%)**			
		**G**_**1**_	**G**_**40**_	**Increased gain(%)** ^*^	**ΔF(G**_**0**_ **-G**_**10**_ **)**
n QTL = 100					
	RR_w_J_	3.39^a^	52.24^e^	14.23	0.050^ab^
	RR_w_D0.2_	3.38^a^	53.47^de^	16.90	0.047^bc^
	RR_w_D0.05_	3.60^a^	55.83^cd^	22.06	0.046^d^
	BL_w_J_	3.25^a^	56.86^bc^	11.36	0.037^e^
	BL_w_D0.2_	3.28^a^	59.55^ab^	16.63	0.035^ef^
	BL_w_D0.05_	3.31^a^	60.58^a^	18.64	0.033^f^
n QTL = 400					
	RR_w_J_	1.93^a^	32.37^d^	24.31	0.052^ab^
	RR_w_D0.2_	1.84^a^	33.04^cd^	26.88	0.048^bc^
	RR_w_D0.05_	1.76^a^	34.06^c^	30.80	0.045^d^
	BL_w_J_	1.62^a^	35.52^b^	14.47	0.038^e^
	BL_w_D0.2_	1.65^a^	37.01^ab^	19.27	0.035^ef^
	BL_w_D0.05_	1.70^a^	37.51^a^	20.88	0.034^f^
n QTL = 2000					
	RR_w_J_	0.81^a^	15.60^c^	23.91	0.050^ab^
	RR_w_D0.2_	0.80^ab^	15.96^c^	26.77	0.049^bc^
	RR_w_D0.05_	0.77^ab^	16.17^c^	28.44	0.044^d^
	BL_w_J_	0.73^ab^	17.47^b^	13.52	0.037^e^
	BL_w_D0.2_	0.73^ab^	18.47^a^	19.75	0.036^ef^
	BL_w_D0.05_	0.65^b^	18.48^a^	20.08	0.034^f^

Different superscripts in the same column show significant differences (p < 0.05); standard errors for all means are for the long-term genetic gain are < 0.21% for G_1_, < 0.79% for G_15_ and for <0.01 ΔF; ^*^the increase in *Rs* from unweighted GP relative to weighting GP under the same prediction model.

The weighting of rare alleles also affects the short-term rate of inbreeding (Tables 3 and 4). For instance, with TH40, weighting with JW [13] decreased the rate of inbreeding by ~ 15%, while using DW decreased the rate of inbreeding a little more. This effect of weighting on inbreeding was slightly larger for RR than for BL.

### Correlation between short-term inbreeding and long-term gain

Given the effect of weighted and unweighted GP on short-term inbreeding and long-term genetic gain, we also examined the correlation between them. The inbreeding coefficient in G_10_ was used as the short-term level of inbreeding and *R*_*s*_ at G_40_ was used as the long-term genetic gain. Across 100 replicates for all eight selection criteria, correlations between the short-term inbreeding and *R*_*s*_ were negative and ranged from −0.65 to −0.44. More QTL led to a higher correlation.

### Genetic variance, accuracy, and loss of alleles

**Figure 4:**
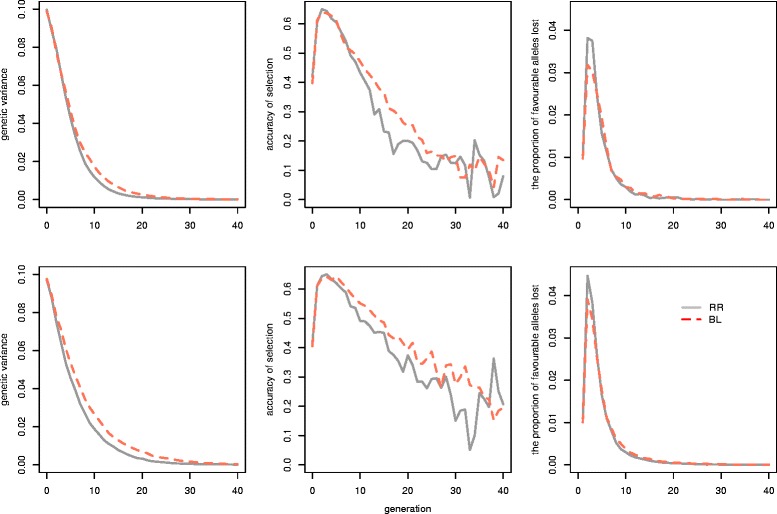
**Change in the mean TBV, accuracy of GEBV and proportion of favourable QTL alleles lost per generation with truncation selection on GEBV estimated with unweighted RR or BL.** The plots in the first row present the results with 100 QTL and those in the second row with 2000 QTL.

To understand why BL was superior to RR for long-term genetic gain, we examined the genetic variance, accuracy of selection and loss of favourable alleles. Figure 4 shows that BL maintained more genetic variance as a consequence of an initial smaller loss of favourable alleles and a higher accuracy of selection, resulting from higher genetic variance. Furthermore, the genetic variance decayed more slowly as the number of QTL increased, but still approached 0 from G_25_ onwards for selection using RR or BL in all scenarios. This explains why the genetic response to selection from G_15_ to G_40_ was smaller than that from G_1_ to G_15_ (Table 2). Furthermore, a larger number of QTL led to a more stable reduction in accuracy of predicted GEBV, but at the same time also led to a larger loss of favourable alleles for both criteria. Table 5 shows the MSD at G_0_ to G_15_ from RR and BL. In all scenarios, BL had a significantly ~7% higher MSD compared to RR.

**Table 5:** **Mendelian selection differential from truncation selection on GEBV**

**Scenario**	**Selection**	**Mendelian sampling differential**
	**Criteria**	**G**_**0**_ **-G**_**15**_
n QTL = 100	RR	0.54^e^
	BL	0.58^d^
n QTL = 400	RR	0.63^c^
	BL	0.66^b^
n QTL = 2000	RR	0.65^bc^
	BL	0.70^a^

Different superscripts in the same column show significant differences (p < 0.05); standard errors for all the means range from 0.006 to 0.009.

**Figure 5:**
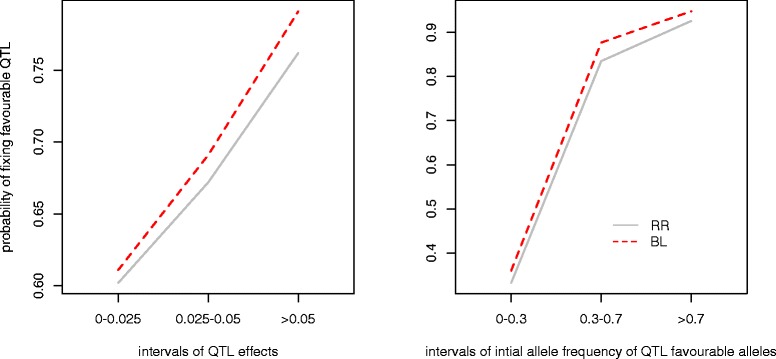
**Probability of a favourable QTL allele being fixed (to a frequency of 1) given its effect and frequency in the scenario** ***100QTL_TH15.*** The two plots show the probability of fixing favourable alleles at the QTL according to its effects or initial favourable allele frequency using unweighted RR or BL.

To further investigate how the prediction models affect the favourable alleles in a long-term selection scheme, we explored the fixation of QTL given their effect and initial favourable allele frequency (Figure 5). These results show that more QTL with a large effect were fixed with BL than with RR, as expected. BL also performed slightly better in terms of fixation of QTL with a small effect or of QTL with low or intermediate initial frequencies of the favourable allele.

**Figure 6:**
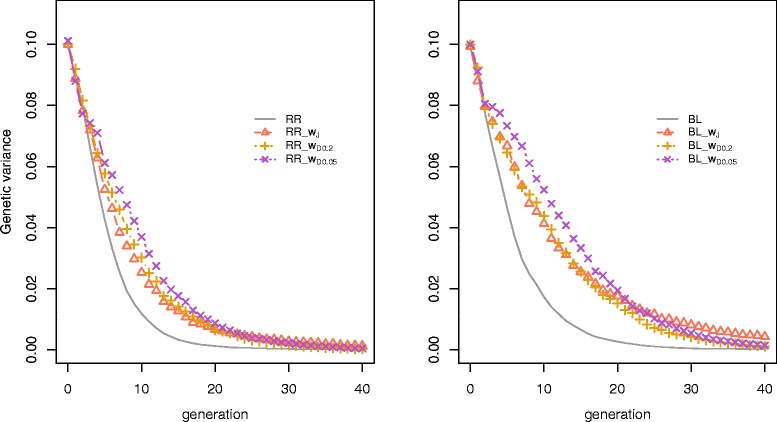
**Genetic variance in the scenario** ***100QTL_TH15.***

To understand how weighted GP resulted in greater long-term gain than unweighted GP, the genetic variance maintained at each generation was investigated. Weighted GP maintained genetic variance in later generations by sacrificing initial genetic gain, and therefore increased the selection limit (Figure 6). Relative to DW, JW maintained a higher genetic variance, but it approached 0 towards the end of the time horizon.

## Discussion

### Dynamic weighting

We have described a novel genomic selection method to maintain genetic variance and increase long-term genetic gain. This method is built upon Jannink’s weighting method [13], in which low-frequency favourable alleles obtain a high weight. The difference with Goddard’s method [12] is that both allele frequencies and marker effects are included in the selection criterion used by Jannink [13], because when it is necessary to estimate many marker effects, these are uncertain and thus whether an allele is favourable or not is also uncertain. Jannink’s weighting method was proven to be successful in boosting the long-term genetic gain compared to unweighted GP. Two aspects are further incorporated in the dynamic weighting method we developed herein. First, it initially puts more weight on low-frequency alleles compared to JW, in order to reduce the chance of losing rare favourable alleles due to genetic drift. Second, it takes the time horizon of the breeding program into account, such that weights on the markers with different allele frequencies become more equal towards the end. After slightly modifying the method by taking these two aspects into account, the long-term genetic gain within a fixed time horizon was higher than with JW. For instance, for TH40, the long-term genetic gain increased by up to 30.8% relative to unweighted GP, and also increased by up to 8% relative to JW, although at the expense of a lower short-term gain.

### Ridge regression vs Bayesian Lasso

Another important finding of our study is that long-term genetic gain depends on the genomic prediction model used. Most studies indicate that prediction accuracies of GEBV obtained with genomic BLUP, which is equivalent to ridge regression, and Bayesian methods, are only slightly different [3,26-28]. These small differences are due to the assumptions made on the distribution of the QTL effects on the trait. In our study, we found that, with BL, accuracy and genetic gain were slightly lower or equal in the first few generations than with RR. However, over a longer time horizon, BL was markedly superior to RR due to genetic variance being maintained. One explanation is that with RR there is more shrinkage towards 0 for rare alleles (although they show a larger effect) [5]. Figure 5 shows that more favourable alleles with low frequencies were lost with RR than with BL. Another explanation is associated with the difference in rate of inbreeding between RR and BL. The higher early rate of inbreeding with RR may be due to a higher probability of co-selecting relatives as parents. We also found that MSD was higher with BL than with RR within 15 generations, which means that BL better captured the within-family variance. This is in line with the study by Habier et al. [29], who showed that the impact of genetic relationships on accuracy was greater for RR than for Bayes-B, while Bayes-B used more information on LD to reach a high accuracy. Moreover, our study shows that a larger number of favourable QTL-alleles were lost and that the genetic variance and accuracy of selection decreased faster with RR, which indicates that RR resulted in greater genetic drift than BL. A strong influence of early inbreeding and genetic drift is seen in the long-term genetic gain in the form of a negative correlation between the level of inbreeding at G_10_ and genetic gain at G_40_ that ranged from −0.65 to −0.46. This is in agreement with Robertson [30], who stated that the use of information on relatives for the sake of immediate gain in early generations always results in a lower eventual limit of selection.

### The impact of time horizons on long-term genetic gain

In the first implementation of Goddard’s optimization theory [12] by Jannink [13], it was shown that, all other things being equal, unweighted GP resulted in more accurate GEBV than weighted GP using JW. This is because the unweighted GP aims at maximizing genetic gain in the next generation. After one or a few generations, response from weighted GP catches up to that from unweighted GP because of higher genetic variance due to the maintenance of rare favourable alleles. These findings were confirmed by our results (Figure 3). The assumption in JW was that all favourable maker alleles will be fixed eventually. However, fixation of all favourable alleles is difficult or even impossible to achieve, in particular when a large number of QTL with small effects need to be detected. This is because genetic drift causes the loss of favourable alleles, especially those that have a small effect. Although the differential weighting was applied at every generation, we found that the genetic variance at generation 40 was close to 0. Based on the suggestion of Bijma [6], we put higher weight on rare alleles at the starting point, since it prevented early drift within the first 10 generations and showed a lower rate of inbreeding and this further increased the selection limit (Tables 3 and 4; Figure 3). However, when the time horizon was shorter, JW only led to a slightly higher standardized long-term gain when using RR and even lower gain when using BL compared to unweighted GP. It is common in animal breeding studies to observe the benefits over a fixed time horizon, and the optimal solutions for a long-term breeding program are those that maximize the cumulated total genetic value over the planning horizon [31,32]. In DW, a shape parameter α’ in the beta distribution was used to adjust the weighting according to the time horizon, with two values for comparison, i.e., 0.05 and 0.2. With a α’ of 0.05, the rare favourable alleles were better maintained than with a α’ of 0.2, which boosted the selection limit with TH40. However, with TH15 and with a α’ of 0.05, DW tended to sacrifice too much short-term gain, which may not be desirable in a real breeding program. Therefore, the rare favourable alleles became less relevant when the time horizon was relatively short. This also means that DW was better able to take into account the importance of rare favourable alleles by adjusting α’than JW in a selective breeding program.

We made two observations when DW was applied for RR or BL. First, the recovery of long-term genetic gain from reduction of short-term gain relative to unweighted GP was faster with RR than with BL. Second, BL weighted by DW tended to sacrifice more short-term genetic gain compared to RR. These two observations imply that BL might already upweight the rare alleles, with the result that DW overemphasizes the importance of preserving rare favourable alleles. Another reason is that BL showed less genetic drift than RR. Therefore, maintaining favourable alleles using DW was less effective when BL was used to estimate marker effects.

### Impact of genetic models on long-term genetic gain

We also examined the impact of different genetic architectures. Heritability of the trait was low (0.1), because genomic selection is mainly advantageous in situations where the accuracy is low, e.g. for traits with low heritability [33]. It is expected that for traits with a high heritability and a finite number of QTL, faster fixation of QTL might further disadvantage the use of unweighted GP compared to weighted GP. To evaluate this, we performed a simulation with the same settings, except that the heritability was increased to 0.35 and the time horizon was set to 40 generations [See Additional file 1: Figure S1]. With a heritability of 0.35, GP methods and weighting methods showed similar patterns as with a heritability of 0.1, except that the loss of the short-term standardized genetic gain using weighted GP was less when heritability was equal to 0.35 compared to 0.1. This is because with a higher heritability, RR and BL are better at finding the correct favourable alleles, such that there is less weighting of rare unfavourable alleles and therefore less loss of short-term genetic gain.

The number of QTL was also varied in our simulations, since it might influence the accuracy of different prediction models. In contrast to prior expectations, the relative superiority of BL over RR was larger when the number of QTL was larger and long-term response was the criterion for comparison. The results showed that number of QTL mainly affected the loss of favourable alleles and the loss of genetic variance, which was greater with RR than with BL. This may be because with more QTL, the selection pressure on each QTL is smaller, and drift therefore becomes relatively more important. The number of QTL did not affect the rate of inbreeding since, here, rate of inbreeding was measured based on pedigree information only. Pedigree inbreeding is only an expectation of the proportion of the genome that is homozygous by identity-by-descent (IBD). Because of variation around this expectation and genetic hitch-hiking due to selection, pedigree inbreeding may not be a good indicator of the true inbreeding [21]. Future studies should thus focus on the genomic inbreeding, which may reflect the true level of inbreeding.

It should be noted that in our study, the assumed genetic model was simplified to contain only additive effects, and genetic variance was expected to decline due to selection and genetic drift. However, Hallander *et al.* [34] showed that the genetic variance could be sustained or even increase in the presence of non-additive genetic effects. However, in case of epistatic effects, for instance, as the number of loci in both simulation and analysis models increases, the number of possible interactions increases exponentially, and it is more difficult to quantify individual epistatic effects when there are hundreds or thousands of loci involved. Therefore, simulations that consider non-additive effects with a large number of QTL require further understanding of the influence of the quantity and distribution of these effects. Moreover, even when epistatic effects exist, this does not reduce the importance of maintaining genetic variance and rare favourable alleles by weighting methods.

### Breeding plans

It should be noted that the aim of this study was to investigate the main mechanisms that have consequences in long-term selection programs, rather than to provide any concrete guidelines to breeding companies. DW showed a lower accuracy and a lower short-term genetic gain than JW, which may be relevant for practical breeding programs. This applies especially in dairy cattle breeding, for which information on genetic progress of the competitors is easily collected from Interbull reports, which allows comparisons between countries and companies. Given these concerns, it appears that JW and DW methods are more relevant for pig or poultry breeding, since they usually involve closed populations. Also, since pigs and poultry have a shorter generation interval than dairy cattle, long-term selection programs are more relevant. Moreover, another common way of increasing selection limits is to switch the selection rule from truncation selection to optimum contribution selection (OCS). OCS works by optimizing the genetic contribution (i.e. number of matings) of each selection candidate, conditional on EBV and average co-ancestry. By doing so, the genetic gain is expected to be maximized and, at the same time, the rate of inbreeding is restricted. This method has been well studied in dairy cattle, pig and fish breeding and has proven to be promising in terms of long-term genetic gain [35-37]. Thus, it will be worthwhile to compare DW with OCS in future studies. Combining DW with OCS may result in a lower rate of inbreeding and higher genetic gain compared to each method used alone.

## Conclusions

This study shows that without weighting methods, BL is superior to RR in maintaining genetic variance and controlling inbreeding, and therefore can result in higher long-term genetic gain, regardless of the number of QTL affecting the trait and length of the planning horizon. With a larger number of QTL, the relative superiority of BL was more pronounced in terms of both absolute and standardized long-term genetic gain, but the difference in rate of inbreeding remained unchanged. Compared to unweighted genomic prediction, both dynamic weighting and Jannink’s weighting can enhance long-term genetic gain and decrease rate of inbreeding with a time horizon of 40 generations. The long-term genetic gain when using dynamic weighting was up to 30.8% greater than that of unweighted genomic prediction, and also up to 8% greater than Jannink’s weighting, although at the expense of a lower short-term genetic gain. With a time horizon of 15 generations, the long-term genetic gain of dynamic weighting can be guaranteed to be at least as high as that of unweighted genomic prediction, whereas Jannink’s weighting cannot. Therefore, dynamic weighting is a promising method that is expected to result in high long-term genetic gain within a fixed time frame.

## Additional files

Additional file 1: Figure S1. Ratio of standardized long-term genetic gain from weighted GP relative to unweighted GP with a heritability of 0.35. Description: The data provided represent the ratio of standardized long-term genetic gain from weighted GP relative to unweighted GP with a heritability of 0.35 and with a time horizon of 40 generations.
