# Supplementary figures and images for: Upweighting rare favourable alleles increases long-term genetic gain in genomic selection programs
Source: Genet Sel Evol. 2015 Mar 21;47(1):19. doi: 10.1186/s12711-015-0101-0 (PMC4367977; doi:10.1186/s12711-015-0101-0)

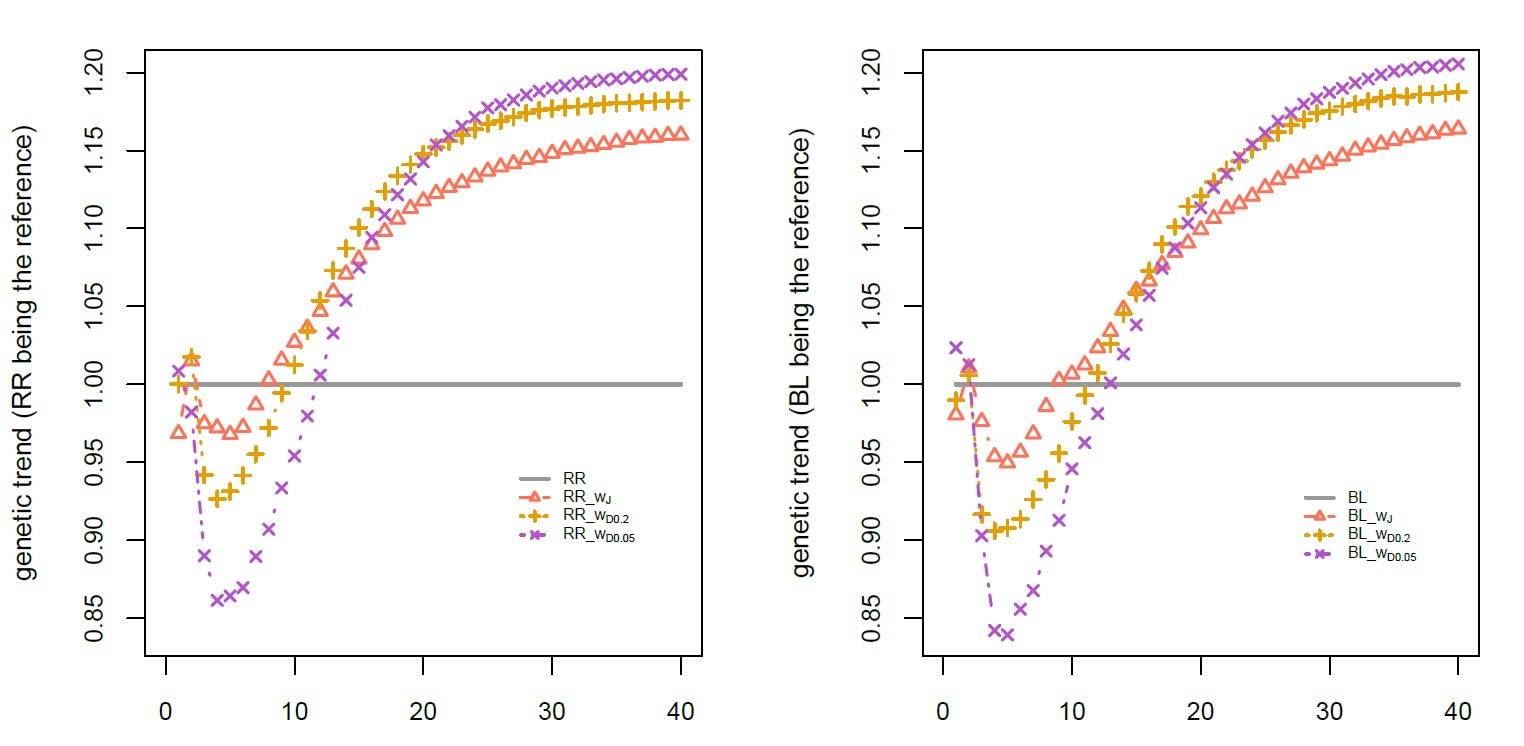

Supplement: Additional file 1: Figure S1. — Ratio of standardized long-term genetic gain from weighted GP relative to unweighted GP with a heritability of 0.35. Description: The data provided represent the ratio of standardized long-term genetic gain from weighted GP relative to unweighted GP with a heritability of 0.35 and with a time horizon of 40 generations. [file 12711_2015_101_MOESM1_ESM.jpeg]
